# TIP – Training of Intensive medical support in the case of a Pandemic

**DOI:** 10.3205/zma001419

**Published:** 2021-01-28

**Authors:** B. Lütcke, J. Günther, J. Köhnen, A. Puchinger, A. Schmidt

**Affiliations:** 1 Universitätsklinikum Erlangen, Klinik für Anästhesiologie, Simulations- und Trainings-Zentrum, Erlangen, Germany; 2 Friedrich-Alexander-Universität Erlangen-Nürnberg, Medizinische Fakultät, SkillsLab PERLE, Erlangen, Germany

**Keywords:** COVID-19, peer-teaching, internal differentiation, self-protection

## Abstract

The COVID-19 pandemic has led to a short-term sharp increase in the demand for auxiliary staff in emergency rooms and intensive care units. Against this background student tutors of the Medical Faculty Erlangen have developed a training concept. The aim was to familiarize students in the clinical section quickly and effectively with skills that are particularly important in a clinical assignment as (student) assistant in the care of corona patients (e.g.: personal protective equipment, intubation assistance, arterial blood collection, assessment of blood gas values and ventilation parameters).

In a blended learning concept, learning materials were prepared in advance and then implemented and deepened in a presence phase. The selection of learning materials and the low supervision ratio (1:2) made it possible to realize an internally differentiated approach. The offer met with great interest among students of all clinical semesters and was evaluated very positively. The skills learned can be applied widely even independently of a pandemic.

## Zusammenfassung

Die COVID-19-Pandemie hat zu einem kurzfristig stark erhöhten Bedarf an Hilfskräften in Notaufnahmen und auf Intensivstationen geführt. Vor diesem Hintergrund haben studentische TutorInnen der Medizinischen Fakultät Erlangen ein Schulungskonzept entwickelt. Ziel war es, Studierende im klinischen Abschnitt schnell und effektiv mit Fertigkeiten vertraut zu machen, die in einem klinischen Einsatz als (studentische) Hilfskraft bei der Betreuung von Corona-Patienten besonders wichtig sind (z.B.: persönliche Schutzausrüstung, Intubationsassistenz, arterielle Blutentnahme, Beurteilung von Blutgaswerten und Beatmungsparametern).

In einem Blended Learning-Konzept wurden Lernmaterialien vorab bearbeitet und in einer Präsenzphase praktisch umgesetzt und vertieft. Durch die Auswahl der Lernmaterialien und den niedrigen Betreuungsschlüssel (1:2) konnte ein binnendifferenzierter Ansatz realisiert werden. Das Angebot stieß auf großes Interesse bei den Studierenden aller klinischen Semester und wurde sehr positiv evaluiert. Die erlernten Fertigkeiten sind auch unabhängig von einer Pandemie breit einsetzbar.

## Introduction

COVID-19 has presented new challenges, especially for medical staff, that were previously little known. Medical knowledge is changing at a speed not previously perceived. Treatment concepts that are still up-to-date are being discarded and replaced in updated form [1].

The aim of the project is to quickly provide medical students of different semesters with pandemic-related basic skills for the care of corona patients for use as assistants. A model for TIP was the deployment of medical students during the polio epidemic in Copenhagen in 1952/53 [2], [3]. TIP recruits medical students from all semesters, so that the level of education of the participants is very inhomogeneous. In addition, practical skills are imparted very inhomogeneously during the various stages of the study program (nursing internship, clinical traineeships, internships, PJ).

## Project description

Against this background, the student tutors of the Skills Lab PERLE (**P**raxis **ER**fahren und **LE**rnen) and the Simulation and Training Center Anaesthesia (STZ) developed a basic training concept for students. The goal is to be able to use it in the short term to support patient care in the context of a pandemic. The focus of this training concept is on skills that are needed in the current situation in the emergency room and intensive care unit. The skills imparted are of course also helpful outside of a pandemic, for example in identifying critically ill patients. 

The student tutors, in cooperation with intensive care personnel, identified 8 skills that can be important for support and are important for the self-protection of staff. These skills are taught in the medical curriculum mainly theoretically and not systematically.

TIP (**T**raining of** I**ntensive Medical Support in the Case of a **P**andemic) is based on a blended learning concept in which the course participants work through learning materials in advance in order to apply and deepen these contents in the practical attendance phase. For the practical training, the participants receive a decision and memory aid (smock pocket card) to focus on learning and practicing practical skills and to support the development of procedural knowledge. A further aspect in the development of the course concept lies in a time-optimized, qualitatively excellent attendance phase.

## Methods

In the preliminary learning module, students deal with various topics:

- **Current COVID-19 information: ***Focus on infection path, incubation period, symptoms, diagnostics, complications, therapy*
- **Ventilation parameters: ***Focus on tidal volume, AMV, etCO2, PIP, PEEP, BIPAP, ventilation in ARDS*
- **Blood gas analysis:*** Focus on standard values*

For a more in-depth study of the topics, further specialist literature is suggested and the reader is encouraged to conduct his own research. These measures are intended to achieve a more homogeneous level of knowledge at the beginning of the practical training of students from different semesters in the sense of an internally differentiated approach [4].

The practical training takes place at 6 stations and lasts four hours. Two participants are trained at each station in rotation by an instructor/tutor. The observance of the distance rules can therefore be implemented without any problems. 

The **basic hygienic aspects** (social distance, mouth and nose protection, hand disinfection) are pointed out during the introduction to the training course **(station 0)**. Throughout the entire training, the tutors also pay attention to the correct implementation of these aspects.

The following skills are taught at the other stations:

- **Protective clothing,** donning and doffing (= “donning/doffing” – with and without buddy)
- **Intubation assistance** (preparation)
- Nasopharyngeal **sample collection** for COVID-19 testing
- **Arterial blood collection **from arterial catheter
- **Arterial puncture** on the puncture trainer
- Handling of **perfusers **and preparation of catecholamines (dilution)
- Basics of **ventilation **and blood gas analysis (alarm station; recognition of critical situations)

**Figure 1:**
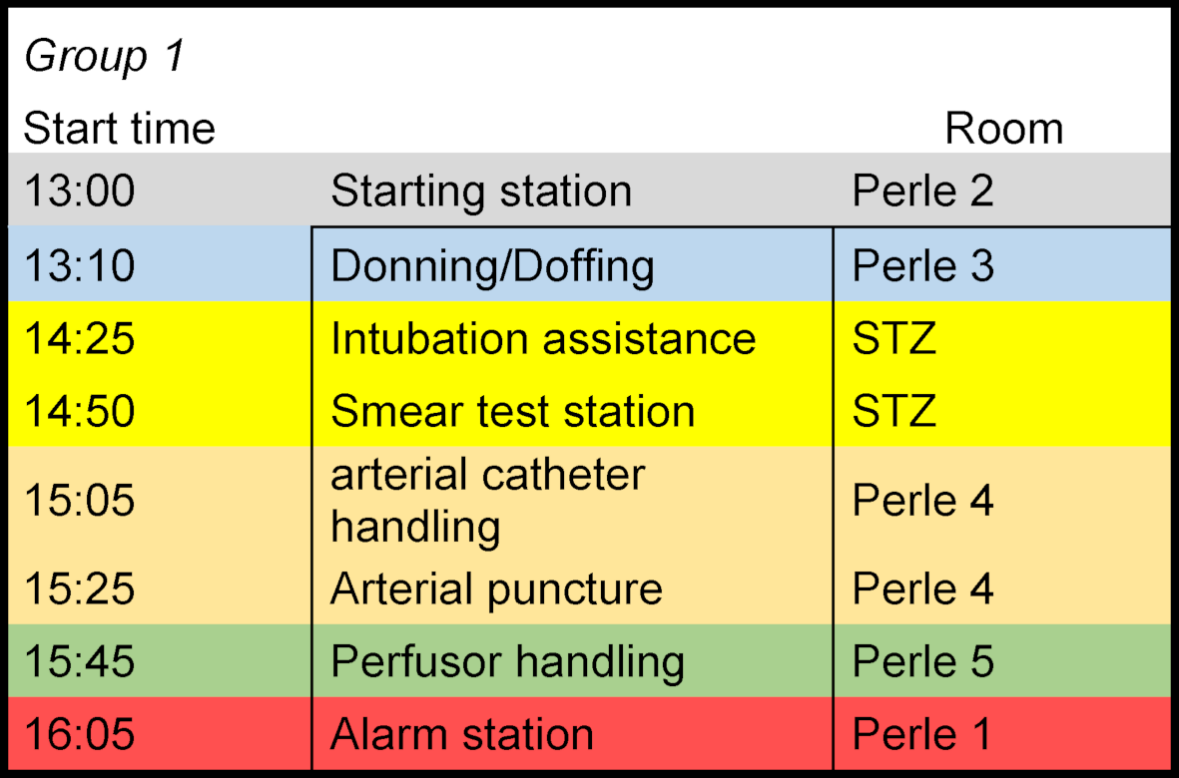
Rotation plan for a group of two

At the wards (see figure 1 (Fig. 1)), previous knowledge is first of all assessed by the tutors who have been comprehensively trained by the intensive care personnel. The skills are then imparted in a differentiated manner within the hospital; students with less previous knowledge receive a basic training, students with more previous knowledge receive more detailed information on the skills. All have the opportunity to deepen their practical skills through multiple practice. In addition to the verbal teaching, film sequences and picture series and elements of the 4-Step-Aproach [5] are used.

The smock pocket card can be used as a decision and memory aid during the training and contains information and tips on

- Putting on and taking off protective clothing
- Blood collection from arterial catheter
- Blood gas analysis
- Arterial puncture
- Intubation Assistance
- Preparation of catecholamines (uptake/concentrations)

## Implementation

**Figure 2:**
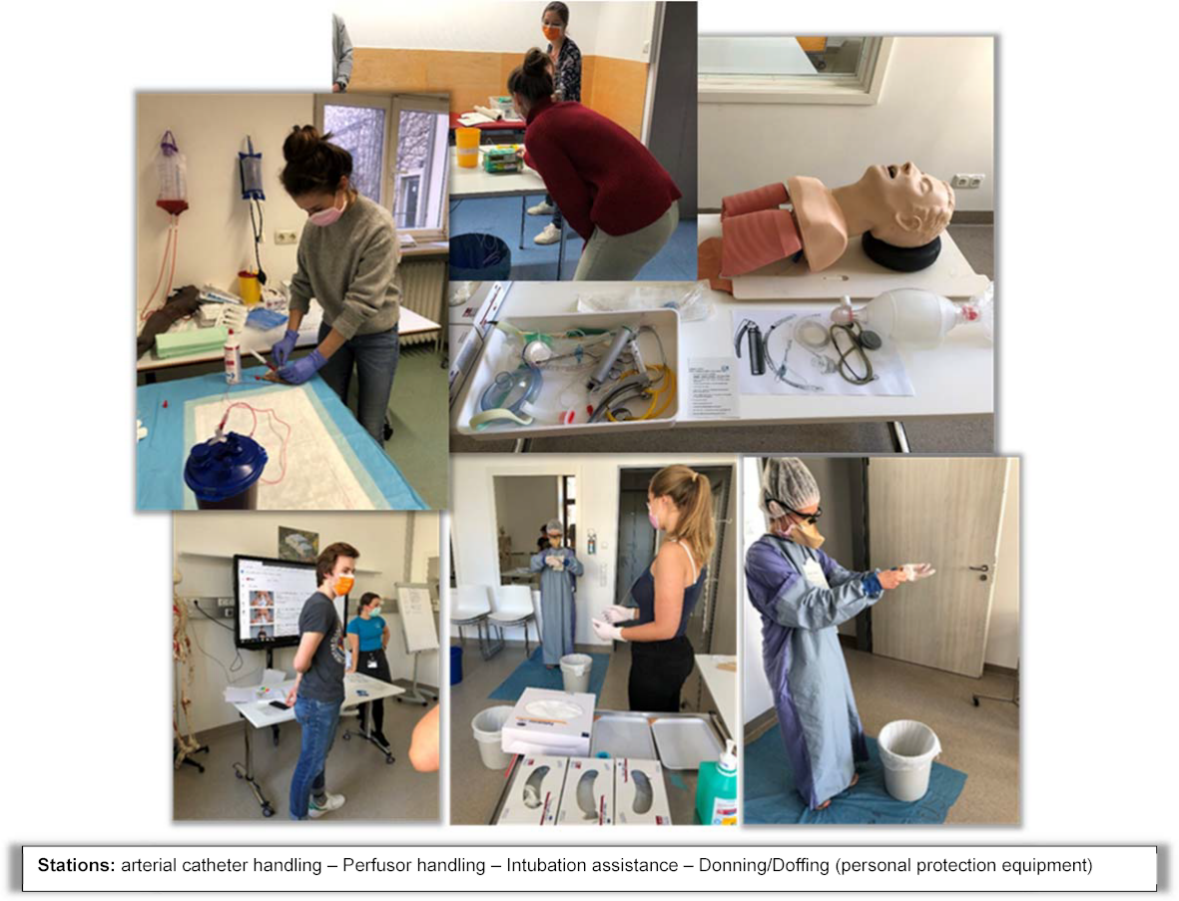
Different stages of the course

On 12 course days 72 students were trained with “TIP” (see figure 2 (Fig. 2)). All participants reflected their impressions in a freely formulated feedback (see table 1 (Tab. 1)). The tutors also presented their experiences and impressions in a feedback. The feedback was used to optimize the course process and the course content. In summary, the choice of stations and content was very positively received. Both the preparation for the course and the implementation at the respective stations was experienced as goal-oriented. The hygiene aspects and the associated self-protection were perceived as important contents, also with regard to further infection situations or for the care of patients hygienically isolated for other reasons. A review of how the trained skills were applied to support intensive care personnel in their practical work has not yet been possible. Due to the tense overall situation, the primary concern was to evaluate the feasibility of the concept.

## Discussion and summary

Due to the low number of COVID-19 patients at the University Hospital Erlangen during the pandemic situation, TIP could not show its full range of services. The current situation and the resulting training program show that aspects of self-protection and hygiene need to be better represented and practically trained in medical studies. Even outside of a pandemic situation, the skills trained in TIP are essential skills in everyday student and professional life. For example, contact with isolated patients (AER, MRSA) in clinical trials and other clinical assignments is not uncommon. The correct use of personal protective equipment protects against the spread of critical germs. 

With the course concept a possibility was created to impart skills in PEER-teaching and to relieve intensive care personnel from training measures for support staff.

## Competing interests

The authors declare that they have no competing interests. 

## Figures and Tables

**Table 1:**
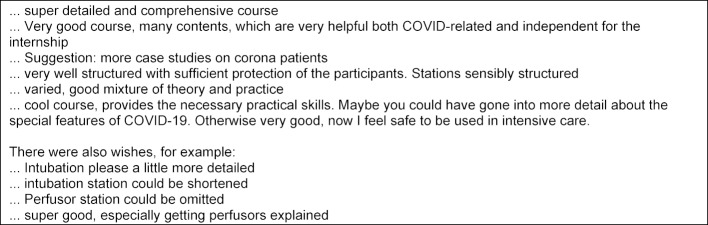
Some extracts from the written participant feedback
